# Consumer acceptability of gluten‐free cookies containing raw cooked and germinated pinto bean flours

**DOI:** 10.1002/fsn3.531

**Published:** 2017-11-20

**Authors:** Courtney Wayne Simons, Clifford Hall

**Affiliations:** ^1^ Wright State University, Lake Campus Celina OH USA; ^2^ North Dakota State University Fargo ND USA

**Keywords:** cookies, germination, pinto bean, sensory evaluation

## Abstract

Beany and grassy flavors in raw edible bean flours reduce consumer acceptability of bean‐based baked products. In order to improve consumer acceptability, beans may be further processed by cooking and germination. However, these operations drive up the cost of end‐products. Therefore, it is necessary to develop formulations, using raw edible bean flours that have acceptable sensory attributes. In this study, cooked, germinated, and germinated/steam‐blanched (GSB) pinto bean flours were used to make gluten‐free cookies, and their sensory characteristics evaluated to determine how their consumer acceptability scores compared. Taste panelists (31) graded cookies made from raw pinto beans with an overall value of 6 on a 9‐point hedonic scale (*p* < .05). This rating was not significantly different from cookies formulated with germinated and GSB flours. Therefore, gluten‐free cookies can be made using raw pinto bean flours at a 40% inclusion level, with similar sensory characteristics as those prepared with flours treated by cooking and germination. Instrumental measurement of cookie hardness and color showed no significant difference in hardness, but significant differences in color. The germinated bean flour produced cookies with a significantly lower L* value and significantly higher a*, b*, Chroma and hue values compared to the other treatments. There was no significant difference in the cookie spread ratio. Proximate composition, water absorption index (WAI), water solubility index (WSI) and gelatinization properties of the flour treatments were characterized.

## 1 INTRODUCTION

The health benefits of pulses have been well documented. These benefits include high protein, high fiber, low fat, significant amounts of vitamins and minerals, and low glycemic index (Brand‐Miller, Dickinson, Barclay, & Celermajer, 2007; Campos‐Vega, Loarca‐Pina, & Oomah, 2010; Derbyshire, 2011; Finley, Burrell, & Reeves, 2007; Jenkins et al., 2012; Tharanathan & Mahadevamma, 2003; Tosh & Yada, 2010). Therefore, regular consumption of pulses may contribute to improvement in health and well‐being. In order to better distribute these benefits, pulse flours may be added to foods such as baked goods, meat, pasta, and extruded snacks. However, direct addition of raw pulse flours has been limited by their beany and other off‐flavors associated with several flavor compounds including n‐hexanal, 3‐cis‐hexenal, 2‐pentyl‐furans, 1‐penten‐3‐one, n‐pentanol, n‐hexanol, n‐heptanol, l‐octen‐3‐ol, trans,trans‐2,4‐nonadienal, trans,trans‐2,4‐decadienal, trans‐2‐nonenal, trans,cis‐2,4‐nonadienal, butyric acid, 2‐methyl butyric acid methyl ester, 2‐pentyl‐pyridine, pentanal, and acetophenone (Boatright & Crum, 1997; Boatright & Lei, 1999; Hsieh, Huang, & Chang, 1982; Sessa & Rackis, 1977).

In order to control these off‐flavors, and hence improve sensory quality, raw bean flours may be heat‐treated or germinated before adding to baked products. For example, Siddiq, Kelkar, Harte, Dolan, and Nyombaire (2013) found that extrusion cooking of pinto and navy bean flours significantly reduced “beany” flavor to the extent that taste panelists were not able to detect it in cookies made with the flours. Shin, Kim, and Kim (2013) prepared soybean flours for bread making by first treating the beans using germination, steaming and roasting techniques. They observed that beany flavor in bread containing germinated bean flours was significantly lower than in bread containing raw soybean flour. They found an even more significant reduction in beany flavor when soybeans were heat treated.

In spite of their utility in reducing unacceptable flavors, extrusion and bean germination processing are likely to add to the cost of pulse flour ingredients and contribute to higher end product prices due to longer processing time, increased energy consumption, labor and other inputs used in making the flours. Therefore, innovating acceptable formulations, using raw pulse flours will make end product prices more affordable and attractive to consumers looking for healthier snack options. This will be especially true for the gluten‐free market where products are already being priced at a premium (Konig, 2015; Singh & Whelan, 2011; Stevens & Rashid, 2008). The gluten‐free market is lucrative for pulse ingredient sales, given the size and growth of the market. At a rate of 10.2% annual growth, the market is expected to reach $6,206 million by 2018 (Missbach et al., 2001). This is a tremendous opportunity since Missbach et al. (2001) found that gluten‐free products show critical nutrient deficiencies. These deficiencies can be addressed by including pulses in gluten‐free products.

In this study raw, cooked, germinated, and germinated/steam‐blanched (GSB) pinto bean flours were used to make gluten‐free cookies, and their sensory properties investigated. Several researchers have studied the effect of adding pulse ingredients to gluten‐free cookies. Pulse ingredients added to gluten‐free cookies so far include germinated/extruded black bean cotyledon flour, extruded navy bean flour, extruded pinto bean flour, commercially available yellow pea flour, dehulled green lentil flour, raw navy bean flour, raw pinto bean flour, precooked and dehulled soybean flour, commercially available soybean flour, raw lupine flour, extruded black bean flour, dehulled pigeon pea flour, and chickpea flour (Bassinello et al., 2011; Maghaydah, Abdul‐Hussain, Ajo, Tawalbeh, & Elsahoryi, 2013; Man, Paucean, & Muste, 2014; Okpala & Chinyelu, 2011; de la Rosa‐Millán, Pérez‐Carrillo, & Guajardo‐Flores, 2017; Siddiq et al., 2013; Tharise, Julianti, & Nurminah, 2014; Yamsaengsung, Berghofer, & Schoenlechner, 2012; Zucco, Borsuk, & Arntfield, 2011). Only three of these studies measured consumer acceptability of gluten‐free cookies made with raw pulse flours (Maghaydah et al., 2013; Okpala & Chinyelu, 2011; Yamsaengsung et al., 2012). Overall acceptability varied from 1.4 to 8.3 on a 9‐point scale, depending on the flour type and concentration. In general, sensory quality declined as the concentration of pulse flour went up. Only one study was found that investigated production of cookies, using raw pinto bean flour. However, a sensory evaluation was not conducted (Zucco et al., 2011). A study by Siddiq et al. (2013) conducted sensory evaluation after making cookies from pinto beans. However, the flour was made from extruded and steam‐cooked beans. No study to date has been reported on sensory quality of cookies using germinated pinto beans. Inclusion of germinated bean flours was of interest since germination has been shown to not only improve sensory quality, but also increase bioavailability of nutrients (Gujral, Angurala, Sharma, & Singh, 2011; López‐Martínez, Leyva‐López, Gutiérrez‐Grijalva, & Heredia, 2017; Luo & Xie, 2013; Urbano et al., 2005). Pinto bean was selected in this study because of its market availability and popularity. USDA–NASS 2016 agricultural statistics data showed that pinto bean was the most popular dry bean in terms of total production compared to others beans. This most recent statistic shows that the pinto bean represented 33% of total dry bean production (cwt) in the US ahead of chickpea (19.0%), black bean (15.2%), navy bean (12.2%), kidney (5.5%) and other dry beans (14.5%) (https://www.nass.usda.gov).

## 2 MATERIALS AND METHODS

Pre‐packaged bags of pinto beans, distributed by Northern Feed and Bean Company (Lucerne, CO) were purchased from Ruler Foods (Celina, OH). Oats, rice, tapioca, and quinoa flours, distributed by Bob's Red Mill (Milwaukie, OR), were purchased from Kroger (St. Marys, OH). All other ingredients, i.e., sugar, butter, egg, xanthan gum, salt, and vanilla were purchased from Walmart Stores Inc. (Celina, OH).

### 2.1 Flour and cookie mix preparation

To prepare raw flour, 1 kg of pinto bean was milled, using a Retsch centrifugal mill (Verder Scientific Inc., Newtown, PA) running at 10,000 rpm and fitted with a 0.5 mm sieve. The flour was collected in Ziploc® bags and stored at room temperature until ready for flour mix preparation. Cooked bean flour was prepared by rinsing 1 kg of pinto beans under running tap water followed by soaking in excess distilled water for 12 hours at room temperature in a Sterilite^®^ polypropylene tub (35.6 cm × 20.3 cm × 12.4 cm). After soaking, the beans were drained to remove excess water and then boiled in fresh excess distilled water for 20 minutes in a stainless steel pot heated on a gas kitchen range. Cooking time started when the water temperature reached of 99°C. The cooked beans were then dried in a convection oven (VWR International, Radnor, PA) at 85°C for 14 hours. After drying, the beans were milled, collected in Ziploc^®^ bags, and stored as previously described. Germinated bean flours were prepared by rinsing 1 kg of pinto beans under running tap water and then steeping them for 20 hours at room temperature in a Sterilite^®^ polypropylene tub (35.6 cm × 20.3 cm × 12.4 cm). The beans were aerated during steeping, using an Aqua Culture^®^ aquarium pump (1,200 ml/min) (Walmart Stores Inc., Bentonville, AR). After steeping, beans were rinsed with distilled water, drained and then placed in a closed cupboard to germinate for 48 hours. The beans were sprayed with a layer of fresh distilled water every 12 hours to ensure that they did not dry out during germination. The beans were then dried, milled, and stored as previously described. GSB bean flours were prepared according to the previously described germination procedure and then steam‐blanched in a steam pot for 25 minutes on a gas kitchen range. Blanching time started when the center temperature of the bean batch reached 93°C. The beans were finally dried and milled as previously described, and held at room temperature until flour analysis and cookie mix preparation. Four cookie flour mixes were made by homogenously combining 40% pinto bean flour (raw, cooked, germinated or GSB flour), 30% oats, 15% rice, 7.5% tapioca, and 7.5% quinoa in 3.8 L PETE plastic jars.

### 2.2 Cookie baking

Two separate batches of cookies, representing each pinto bean flour treatment were prepared in order to replicate cookie dough formulation and baking procedure. Baking protocol proceeded by combining and creaming sugar and butter (Table 1) for 3 minutes, using a Dash Go^®^ kitchen mixer (Walmart Stores Inc., Bentonville, AR) operating at speed 1. Egg and vanilla were gradually added while mixing for another 4 minutes at speed 1 to ensure that all the cream was combined with the egg. Salt and xanthan gum were added to the cookie flour mix and the mix then manually folded in, using a spatula to achieve a soft homogenous dough. The dough was transferred to a 3.8 L Ziploc^®^ bag and refrigerated for 1 hour to harden. After hardening, the dough was sheeted, using a rolling pin on a counter top lined with parchment paper. Dowel rods (0.5 cm × 22 cm) were used to ensure that the cookie dough sheet maintained consistent thickness. Cookies were then cut from the sheet, using a 36 mm diameter nonserrated circular cookie cutter, and transferred to a 40.6 cm × 30.5 cm × 2.54 nonstick metal baking tin (Wilton Industries Inc, Woodridge IL). The cookies were then baked at 190.6°C for 7 minutes after which the cookies were removed from the oven and allowed to cool for 2 minutes on the baking tray. Immediately afterwards, the cookies were transferred to a kitchen countertop lined with grease paper where they were allowed to finish cooling. The prepared cookie dough was large enough to bake four batches of cookies with each tray holding 20 cookies (yield = 80 cookies). However, only one tray was placed in the oven at a time to ensure that the tray was placed in the same position in each run, and that the oven temperature variation was minimized.

**Table 1:** Cookie formulation

Ingredient	Percentage (%)
Flour mix^a^	35.5
Sugar	29.6
Butter	26.0
Whole egg	7.1
Xanthan gum	1.1
Salt	0.2
Vanilla	0.6

a Contains the following flours: pinto bean (40%), oats (30%), rice (15%), tapioca (7.5% and quinoa (7.5%).

### 2.3 Chemical and functional properties of flour treatments

All chemical and functional properties were determined in triplicate unless otherwise indicated. Moisture content was determined based on AOAC Method 925.10 (AOAC, 1990). Ash content was determined following AACC Method 08–01.01 (AACCI, 2011). Total lipids were determined based on method adapted from AOCS methods Af 3‐53, Am 2‐93, and Aa 4‐38 (AACCI, 2011). Round‐bottom flasks (250 mL) were preweighed. Bean flour samples (5 g) were weighed and collected in a folded 125 mm diameter filter paper (Whatman No. 1 qualitative, VWR International, Radnor, PA) and transferred into a thimble holder. Lipids were extracted with hexane for 12 hours with a Soxhlet apparatus (Glas‐Col^®^ combo mantle, Terre Haute, IN). Hexane was evaporated with a rotary evaporator (Yamato RE 200 with Yamato BM 100 water bath, Yamato Scientific Co. Ltd., Japan) at 40°C under vacuum. Flasks were then reweighed to determine increase in weight. Weight difference was used to calculate percentage of total lipids extracted. Total protein was determined in duplicate, using a nitrogen combustion analyzer (Leco FP‐628, St. Joseph, MI) according to AACC International Approved Method 46‐30.01(AACCI, 2011). Total and resistant starch (TS) was determined in duplicate, using Megazyme‐resistant starch kit (Megazyme International, Bray, Ireland). The total starch was calculated as the sum of the resistant and soluble starch. Water absorption index (WAI) and water solubility index (WSI) were determined based on a modified method of Anderson (1982). Pinto bean flours (<210 μm, 2.5 g) were added to 45 mL centrifuge tubes containing magnetic stir bars. Distilled water (30 mL) was added, and the tubes were sealed and vigorously agitated to break lumps. Tubes were placed on a magnetic stirrer, mixed for 30 min, and then centrifuged at 3,000 rpm, using a Beckman Coulter Allegra ×14 centrifuge (Pasadena, CA). The supernatant was decanted and the container weighed. The weight of sediment was determined by difference. The supernatant collected, was placed in a crucible and dried in a convection oven at 100°C overnight. WAI and WSI were calculated from equations 1 and 2, respectively:

(1) $$\mathrm{WAI}\;=\;\frac{\mathrm{weight}\;\mathrm{of}\;\mathrm{the}\;\mathrm{wet}\;\mathrm{sediment}(g)}{\mathrm{initial}\;\mathrm{weight}\;\mathrm{of}\;\mathrm{the}\;\mathrm{dry}\;\mathrm{flour}(g)}$$

(2) $$\mathrm{WSI}\;=\;\frac{\mathrm{weight}\;\mathrm{of}\;\mathrm{the}\;\mathrm{wet}\;\mathrm{sediment}(g)}{\mathrm{initial}\;\mathrm{weight}\;\mathrm{of}\;\mathrm{the}\;\mathrm{dry}\;\mathrm{flour}(g)}\;\times \;100$$

Pasting properties were determined, using a Newport Scientific Rapid Visco‐Analyzer (RVA) based on approved AACCI Method 76‐21.01 (AACCI, 2011).

### 2.4 Cookie evaluation

The spread ratio was determined by measuring the width and thickness of 10 randomly selected cookies from each baking run (n = 20), and then dividing the width by the thickness. Each of the randomly selected cookies from the spread ratio test was also measured for texture using a CT3 texture analyzer (Brookfield, Middleboro, MA) equipped with a three‐point bend assembly. The instrument was set at a speed of 1 mm/sec; 4 mm target value and 10 g trigger load. The color of the flours was determined, using a CR‐410 Konica Minolta Chroma meter (Konica Minolta Inc., Japan) and reported as L*, a*, b* values. The hue angle was calculated by finding the ratio of a* to b* and then determining the inverse tangent. The chroma was calculated using the following equation:

$$\mathrm{Chroma}={\left(a^{2}+b^{2}\right)}^{1/2}$$

### 2.5 Sensory evaluation

Sensory evaluation of cookies was based on a hedonic test, using 31 sensory panelists obtained after Institutional Review Board (IRB) human subject research approval. Panelists included students, faculty, and staff over 18 years of age who responded to email invitation to attend the sensory evaluation exercise. There was no criteria in the invitation that they were consumers of gluten‐free products. The evaluation was done in a classroom. Tables were spaced to reduce opportunities for interference by, or communication with other panelists. Nevertheless, they were advised not to communicate during the tasting. Each panelist was provided with four cookies representing each treatment. The cookies were assigned a random three‐digit number and presented in random order to the panelists. They were not otherwise provided with the identity of the cookies, including not telling them that they were gluten‐free. Along with the samples, they were provided with a bottle of drinking water and asked to drink between sampling each cookie, and allow their palate to rest for at least 30 seconds before moving to the next sample. They panelists rated the appearance, flavor, texture and overall acceptability of the cookies on a 9‐point scale (9 = like extremely, 8 = like very much, 7 = like moderately, 6 = like slightly, 5 = neither like nor dislike, 4 = dislike slightly, 3 = dislike moderately, 2 = dislike very much, 1 = dislike extremely).

### 2.6 Statistical analysis

Cookie baking was done on two separate days and the spread ratio, hardness, and color values reported as means of the two baking runs. Analysis of these attributes was based on a completely randomized design. Sensory data was based on a completely randomized block design with panelists representing 31 blocks. SAS software (SAS Institute, Cary, NC) was used to conduct analysis of variance (ANOVA) and comparison of means done, using Tukey's multiple comparison test. Differences were considered significant at *p* ≤ .05.

## 3 RESULTS AND DISCUSSION

### 3.1 Chemical and functional properties

Chemical and functional properties of flour treatments are presented in Table 2. Ash content in cooked bean flours (2.96%) was lower than the amount observed in the other flour treatments. This could be because of leaching of minerals into the cooking water during boiling. The lipid content in the cooked, germinated, and GSB flour was higher than the amount observed in raw flour, suggesting increased hexane extractability of lipids after treatment. Protein content appeared to be relatively stable. This was consistent with a previous study by Simons et al. (2014) that showed that protein content in pinto beans after cooking was generally unaffected. Njintang et al. (2001) evaluated dry red beans and reported that total protein content did not change after germination. Resistant starch reduced from a 29.98% in raw flour to 4.01% and 4.74% in cooked and GSB flour treatments, respectively. Previous studies have also shown significant reduction of resistant starch after heat treatment (Simons, Hall, & Biswas, 2017; Simons, Hall, & Tulbek, 2012; Simons et al., 2014). WAI index increased after cooking and GSB treatments likely due to starch gelatinization. Gelatinized starch has higher water retention properties compared to native starch (Lv, Wu, Wang, Li, & Qin, 2011). The apparent increase in total starch after germination and heat treatment was unexpected. A possible reason could be that modification of starch and reduction of antinutrients during treatment provided greater accessibility of α‐amylase enzymes to the starch digestion assay. WSI of raw flour (27.31%) was much higher than the cooked, germinated and GSB flours. The lower WSI of the cooked flour (10.4%) could be attributed to the leaching of soluble components from the beans such as proteins, sugars, and low‐molecular polysaccharide fractions into the water during cooking. Similarly, GSB flours may have produced a lower WSI (12.49%) due to removal and loss of soluble components during steaming. This loss was evidenced by a change in the steam water color to a reddish‐brown at the end of blanching. The lower WSI in germinated beans (12.49%) is similar to results obtained by Njintang et al. (2001). They found that germinated red bean flours had a WSI of 13.18% when the beans were dried at 80°C. The slightly lower WSI obtained in this study could be due to the higher drying temperature of 85°C that was used. This is consistent with Njintang et al. (2001) finding in their study that WSI decreased with increased drying temperature.

**Table 2:** Chemical and functional properties of pinto bean flours used to prepare gluten‐free cookie flour mix

Treatment	Moisture (%)	Ash (%)	Total lipids (%)	Protein (%)	Resistant starch (%)	Total starch (%)	WAI (g/g)	WSI (%)
Raw	3.99 ± 0.16	4.11 ± 0.06	1.36 ± 0.17	21.31 ± 0.01	29.98 ± 0.95	36.72 ± 1.21	2.51 ± 0.03	27.31 ± 0.46
Cooked	6.15 ± 0.49	2.96 ± 0.06	2.33 ± 0.43	22.76 ± 0.32	4.01 ± 0.52	42.67 ± 1.23	3.09 ± 0.07	10.4 ± 0.06
Germinated	1.97 ± 0.35	4.19 ± 0.43	1.69 ± 0.25	22.58 ± 0.03	30.33 ± 0.81	38.00 ± 0.98	2.74 ± 0.01	15.44 ± 0.09
GSB	2.13 ± 0.05	4.12 ± 0.28	1.83 ± 0.19	22.53 ± 0.03	4.74 ± 0.22	41.17 ± 1.00	3.01 ± 0.01	12.49 ± 0.12

Raw and germinated flours had similar pasting characteristics (Table 3) while cooked and GSB flours had similar pasting characteristics. In general, the viscosities of raw and germinated flours were higher compared to cooked and GSB flours. This suggest presence of more native starch in these samples compared to heat‐treated samples since increase in viscosity during starch rapid viscoanalysis (RVA) is associated with absorption of water by native starch, starch swelling, and gelatinization (Delcour & Hoseney, 2010a, 2010b). The lower viscosities observed in cooked and GSB flour treatments are likely because starches in these samples were already partially gelatinized. The lower setback viscosities of the heat‐treated cookies could be an advantage in terms of having lower retrogradation rates, and hence lower cookie staling rates during storage.

**Table 3:** Pasting properties of flour mixes used to make cookie

Treatment	Peak time (min)	Pasting temp. (°C)	Peak viscosity (cP)	Breakdown (cP)	Trough (cP)	Setback (cP)	Final viscosity (cP)
Raw	5.6 ± 0.0	77.9 ± 0.4	1546.5 ± 324.6	286.0 ± 82.0	1260.5 ± 242.5	847.0 ± 158.4	2107.5 ± 400.9
Cooked	5.7 ± 0.0	86.0 ± 0.6	1071.0 ± 19.8	240.0 ± 11.3	831.0 ± 8.5	403.0 ± 5.7	1234.0 ± 14.1
Germinated	5.7 ± 0.0	78.7 ± 0.6	1568.5 ± 19.1	364.0 ± 9.9	1204.5 ± 9.2	781.5 ± 10.6	1986.0 ± 19.8
GSB	5.6 ± 0.0	86.8 ± 0.6	1019.5 ± 4.9	244.5 ± 4.9	775.0 ± 0.0	372.5 ± 2.1	1147.5 ± 2.1

It should be noted here that while many of the chemical and functional attributes shown in Tables 2 and 3 are likely to be significantly different among flour treatments, the authors cannot conclude that they are. This is because the flour formulation step was not treated as statistical experimental units. Rather, for statistical analysis, the cookie formulation/cookie baking, and taste testing were the replicated experimental units. Nevertheless, apparent differences shown, provide clues to differences that may be investigated further.

### 3.2 Physical properties

Spread ratio (Table 4) was in the range of 6.1 to 6.6 and was not significantly different. Other studies incorporating raw pulses in gluten‐free cookies at 40% found a spread ratio ranging from 7.15 to 8.41 (Maghaydah et al., 2013; Okpala & Chinyelu, 2011; Yamsaengsung et al., 2012). Spread ratio is influenced by the water‐holding capacity of the flours used, which is affected by protein content. As total protein increases, spread ratio decreases, due to increased water‐holding, and hence high viscosity resulting in restriction of lateral flow. Other factors at play that may also influence water‐holding and spread are sugar granulation, and type of sugar (Doescher & Hoseney, 1985; Fuhr, 1962; Maghaydah et al., 2013; Yamsaengsung et al., 2012). Cookie hardness was not significantly different among treatments, likely because protein content in pinto bean flours was very similar (Table 1). Although setback viscosities of the heat‐treated flours was lower than the raw and GSB flours (Table 3), the difference was not large enough to produce any significant difference in hardness.

**Table 4:** Spread ratio and texture of gluten‐free cookies

Treatment	Spread ratio	Hardness (g)
Raw	6.6 ± 0.25	2014.0 ± 578.90
Cooked	6.1 ± 0.17	1473.6 ± 102.62
Germinated	6.6 ± 0.06	1892.2 ± 291.01
GSB	6.2 ± 0.23	1643.0 ± 169.49

Spread ratio and hardness of cookies were not significantly different at *p* ≤ .05.

**Figure 1:**
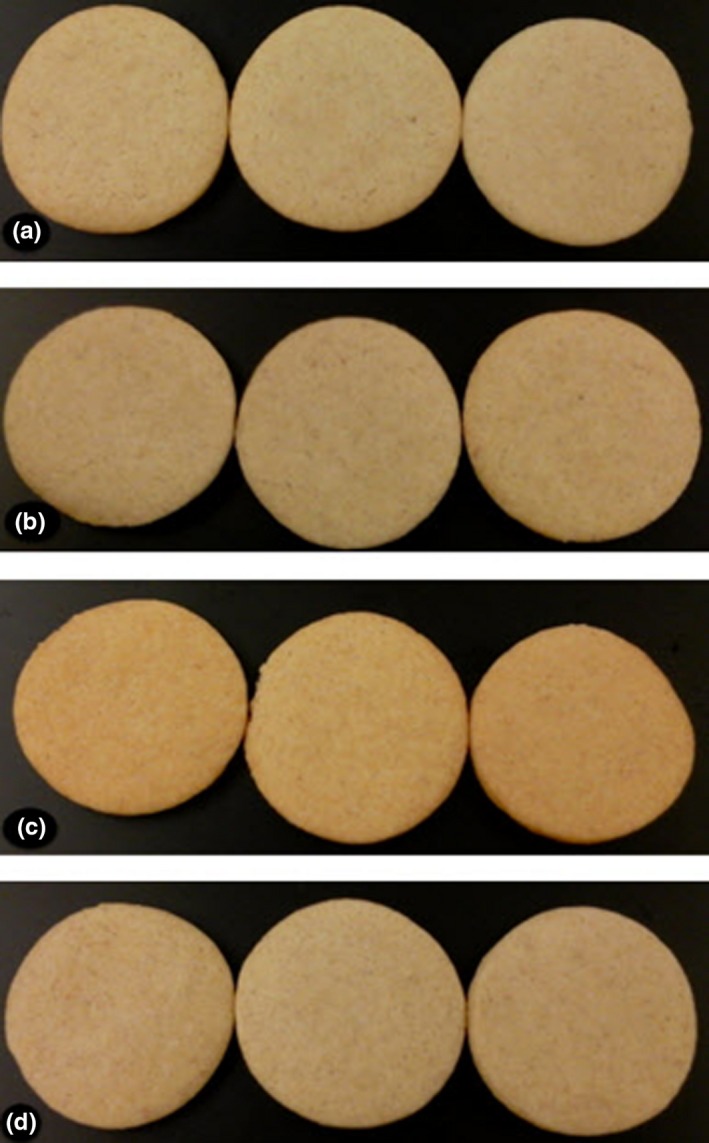
Gluten‐free cookies made with mix containing pinto bean flours. (a) Raw pinto bean flour mix. (b) Cooked pinto bean flour mix. (c) Germinated pinto bean flour mix. (d) Germinated/Blanched pinto bean flour mix. Average dimensions (diameter × height) = 49.8 mm × 7.7 mm

Color measurements showed that cookies made with germinated bean flours had a significantly lower L* value compared to the other flours (Table 5). It also had significantly higher a* and b* values. This corresponded to a red hue (14.15) with a significantly high chroma (31.85) compared to the other treatments. The difference in color characteristics observed in the germinated beans is likely due to greater Maillard browning reactions caused by the presence of more free amino acids and sugars released by enzymes during germination. Cookies made with GSB flour did not reach the same hue and chroma. A likely reason could be a small reduction in reducing sugars and free amino acids caused by water‐leaching during the steam blanching process. However, there was no corresponding reduction in total starch and total proteins after germination. Images of cookies from each treatment are shown in Figure 1. There was a notable absence of a cracked upper surface. Surface cracking is caused by low surface moisture of cookies (Delcour & Hoseney, 2010a, 2010b). The high protein content and their excellent water retention properties are likely contributing to the surface moisture retention and smooth surface.

**Table 5:** Color of gluten‐free cookies

Treatment	L*	a*	b*	Hue angle	Chroma
Raw	73.3 ± 0.82a	3.20 ± 0.32a	26.17 ± 0.50a	6.97 ± 0.62a	26.36 ± 0.52a
Cooked	72.3 ± 0.08a	2.87 ± 0.26a	24.67 ± 0.22b	6.64 ± 0.54a	24.83 ± 0.25b
Germinated	66.8 ± 0.41b	7.79 ± 0.27b	30.89 ± 0.14c	14.15 ± 0.44b	31.85 ± 0.17c
GSB	71.8 ± 0.99a	3.43 ± 0.23a	25.74 ± 0.16a	7.59 ± 0.46a	25.97 ± 0.18a

Means followed by different letters within the same column is significantly different at *p* ≤ .05.

### 3.3 Sensory evaluation

**Figure 2:**
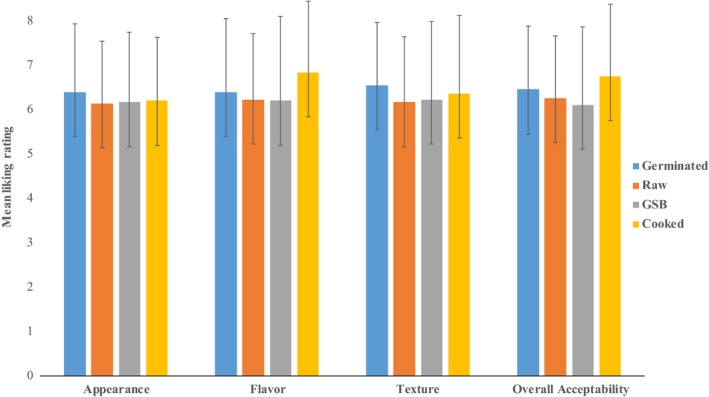
Likeness rating of cookies made with germinated, raw, GSB, and cooked pinto bean flours

In general, taste panelists attributed an acceptability score of 6.0 (liked slightly) for cookies made with each bean flour treatment, with no significant differences between them (Figure 2). Studies done by Okpala and Chinyelu (2011), and Maghaydah et al. (2013) measured consumer acceptability of gluten‐free cookies containing raw pulse flours (lupine and pigeon peas) at the same 40% inclusion. They reported a similar overall acceptability of 6.03 using a 9‐point hedonic scale. Overall, heat treatment and germination did not provide any additional value with respect to consumer acceptability. This could be because the beany flavor in the raw flour was sufficiently masked by the other ingredients including the other gluten‐free flours, butter and sugar. Therefore, this study provides model to demonstrate that with the right combination of ingredients, food manufacturers can create gluten‐free bakery flour mixes and acceptable bakery end products, using raw pulses up to 40%. This would reduce the cost of production that would be incurred if cooked or germinated pulse flours were used.

However, an important question to address is whether or not the inclusion of raw untreated pulse flours in bakery flour mixes at such a high level (40%) will have any large negative effect on the safety of their end products. Raw pulse flours are known to have several antinutrients, including flatulence‐causing α‐galactosides, trypsin inhibitors, chymotrypsin inhibitors, α‐amylase inhibitors, phytates, phytohemagglutinins, tannins, saponins, and oxalates. These antinutrients can be significantly reduced by application of techniques such as soaking, dry heat, fermentation, roasting, boiling, autoclaving, extrusion cooking, microwaving, and germination (Adamidou, Nengas, & Grigorakis, 2011; Chitra, Singh, & Rao, 1996; Frias, Diaz‐Pollan, Urbano, Vidal‐Valverde, & Sotomayor, 2000; Hefnawy, 2011; Marquez & Alonso, 1999; Okpala & Chinyelu, 2011; Sathe, Salunke, & Cheryan, 1984; Simons et al., 2014). Nevertheless, there is limited research in literature to prove or disprove the toxicological safety of pulse‐rich bakery products. One study by Okpala and Chinyelu (2011) investigated antinutrients levels (saponins, oxalates and trypsin inhibitors) in cookies made with pigeon peas and cocoyam. Saponins and trypsin inhibitors were considered to be very low, even at 60% inclusion of raw pigeon pea flour. They suggested that this could be due to soaking of the beans prior to drying and milling. Therefore processors may consider soaking as a necessary step prior to drying and milling raw bean flours for use as ingredients. In their study, Okpala and Chinyelu (2011) also found that oxalates in cookies decreased as the amount of pigeon peas increased, indicating that the oxalates were mainly being contributed by the cocoyam in the formulation and not the pigeon peas. They also found that there was a significant decrease in protein digestibility in the cookies as the pigeon pea flour increased from 20% to 60%. However, they argued that the low trypsin level (0.08 mg/100g) was unlikely to be the cause. They suggested instead that it is likely due to the production of indigestible compounds from the complexing of amino acids and sugars in nonenzymatic browning reactions during baking. Measurement of carbohydrates digestibility in their study showed that there was significant reduction in digestion rate only when the pigeon pea fraction in the composite flour exceeded 40%. This study by Okpala and Chinyelu (2011) is a fair indication that cookies made using presoaked raw bean flours at 40% inclusion level are safe to eat. However, a more comprehensive study of antinutrients in pulse‐based bakery end products including cookies, is needed to provide further support.

## 4 CONCLUSIONS

Sensory evaluation suggest that consumers will find cookies that are made with flours containing 40% raw pinto beans to be acceptable at the same level as cookies made with pretreated pinto beans. Therefore, food manufacturers can reduce the cost of inputs, using raw pinto beans and similar pulses in bakery formulations such as cookies. Further studies are needed to investigate antinutrients in cookies and other bakery products made with raw bean flours. However, current evidence suggests little or no significant risk.

## CONFLICT OF INTEREST

The authors declare that they have no conflict of interest.

## References

[fsn3531-bib-0001] AACCI (2011). Approved methods of the AACC (11th edn). St Paul, MN: American Association of Cereal Chemists International.

[fsn3531-bib-0002] Adamidou, S. , Nengas, I. , & Grigorakis, K. (2011). Chemical composition and antinutritional factors of field peas (Pisum sativum), chickpeas (Cicer arietinum), and faba beans (Vicia faba) as affected by extrusion preconditioning and drying temperatures. Cereal Chemistry, 88, 80–86.

[fsn3531-bib-0003] Anderson, R. A. (1982). Water absorption and solubility and amylograph characteristics of roll‐cooked small grain products. Cereal Chemistry, 59, 265–269.

[fsn3531-bib-0004] AOAC (1990). Official methods of analysis (15th edn). Arlington VA: Association of Official Analytical Chemists.

[fsn3531-bib-0005] Bassinello, P. Z. , Freitas, D. G. , Ascheri, J. R. , Takeiti, C. Y. , Carvalho, R. N. , Koakuzu, S. N. , & Carvalho, A. V. (2011). Characterization of cookies formulated with rice and black bean extruded flours. Procedia Food Science, 1, 1645–1652.

[fsn3531-bib-0006] Boatright, W. L. , & Crum, A. D. (1997). Odor and flavor contribution of 2‐pentyl pyridine to soy protein isolates. Journal of American Oil Chemist's Society, 74, 1575–1581.

[fsn3531-bib-0007] Boatright, W. L. , & Lei, Q. (1999). Compounds contributing to the beany odor of aqueous solutions of soy protein isolates. Journal of Food Science, 64, 667–670.

[fsn3531-bib-0008] Brand‐Miller, J. , Dickinson, S. , Barclay, A. , & Celermajer, D. (2007). The glycemic index and cardiovascular disease risk. Current Atherosclerosis Reports, 9, 479–485. 18377788 10.1007/s11883-007-0064-x

[fsn3531-bib-0009] Campos‐Vega, R. , Loarca‐Pina, G. , & Oomah, B. D. (2010). Minor components of pulses and their potential impact on human health. Food Research International, 43, 461–482.

[fsn3531-bib-0010] Chitra, U. , Singh, U. , & Rao, P. (1996). Phytic acid, in vitro protein digestibility, dietary fiber, and minerals of pulses as influenced by processing methods. Plant Foods for Human Nutrition, 49, 307–316. 8983057 10.1007/BF01091980

[fsn3531-bib-0011] Delcour, J. A. , & Hoseney, R. C. (2010a). Principles of cereal science and technology (pp. 33–39). St. Paul, Minnesita: AACC International Inc..

[fsn3531-bib-0012] Delcour, J. A. , & Hoseney, R. C. (2010b). Principles of cereal science and technology (pp. 216–217). St. Paul, Minnesita: AACC International Inc..

[fsn3531-bib-0013] Derbyshire, E. (2011). The nutritional value of whole pulses and pulse fractions In Tiwari B. K., Gowen A., & McKenna B. (Eds.), Pulse Foods (pp. 363–383). London: Elsevier.

[fsn3531-bib-0014] Doescher, L. C. , & Hoseney, R. C. (1985). Effect of sugar type and flour moisture on surface cracking of sugar‐snap cookies. Cereal Chemistry, 62, 263–266.

[fsn3531-bib-0015] Finley, J. W. , Burrell, J. B. , & Reeves, P. G. (2007). Pinto bean consumption changes SCFA profiles in fecal fermentations, bacterial populations of the lower bowel, and lipid profiles in blood of humans. Journal of Nutrition, 137, 2391–2398. 17951475 10.1093/jn/137.11.2391

[fsn3531-bib-0016] Frias, J. , Diaz‐Pollan, C. , Urbano, G. , Vidal‐Valverde, C. , & Sotomayor, C. (2000). Influence of processing on available carbohydrate content and antinutritional factors of chickpeas. European Food Research and Technology, 210, 340–345.

[fsn3531-bib-0017] Fuhr, F. R. (1962). Cookie spread: Its effect on production and quality. Bakers Digest, 36, 56–60.

[fsn3531-bib-0018] Gujral, H. S. , Angurala, M. , Sharma, P. , & Singh, J. (2011). Phenolic content and antioxidant activity of germinated and cooked pulses. International Journal of Food Properties, 14, 1366–1374.

[fsn3531-bib-0019] Hefnawy, T. (2011). Effect of processing methods on nutritional composition and anti‐nutritional factors in lentils (Lens culinaris). Annals of Agricultural Sciences, 56, 57–61.

[fsn3531-bib-0020] Hsieh, O. A. L. , Huang, A. S. , & Chang, S. S. (1982). Isolation and identification of objectionable volatile flavor compounds in defatted soybean flour. Journal of Food Science, 47, 16–18.

[fsn3531-bib-0021] Jenkins, D. , Kendall, C. , Augustin, L. , Mitchell, S. , Sahye‐Pudaruth, S. , Mejia, S. , & Josse, R. (2012). Effect of legumes as part of a low glycemic index diet on glycemic control and cardiovascular risk factors in type 2 diabetes mellitus a randomized controlled trial. Archives of Intern Medicine, 172, 1653–1660. 10.1001/2013.jamainternmed.70 23089999

[fsn3531-bib-0022] Konig, J. (2015). Gluten‐free food database: The nutritional quality and cost of packaged gluten‐free foods. Peerj, 3, e1337. 26528408 10.7717/peerj.1337 PMC4627916

[fsn3531-bib-0023] López‐Martínez, L. X. , Leyva‐López, N. , Gutiérrez‐Grijalva, E. P. , & Heredia, J. B. (2017). Effect of cooking and germination on bioactive compounds in pulses and their health benefits. Journal of Functional Foods, https://doi.org/10.1016/j.jff.2017.03.002.

[fsn3531-bib-0024] Luo, Y. , & Xie, W. (2013). Effect of germination conditions on phytic acid and polyphenols of Faba bean sprouts (Vicia faba L). Legume Research, 36, 489–495.

[fsn3531-bib-0025] Lv, X. , Wu, L. , Wang, J. , Li, J. , & Qin, Y. (2011). Characterization of water binding and dehydration in gelatinized starch. Journal of Agricultural and Food Chemistry, 59, 256–262. 21126005 10.1021/jf103523u

[fsn3531-bib-0026] Maghaydah, S. , Abdul‐Hussain, S. , Ajo, R. , Tawalbeh, Y. , & Elsahoryi, N. (2013). Effect of lupine flour on baking characteristics of gluten free cookies. Advance Journal of Food Science and Technology, 5, 600–605.

[fsn3531-bib-0027] Man, S. , Paucean, A. , & Muste, S. (2014). Preparation and quality evaluation of gluten‐free biscuits. Bulletin UASVM Food Science and Technology, 71, 38–44.

[fsn3531-bib-0028] Marquez, M. , & Alonso, R. (1999). Inactivation of trypsin inhibitor in chickpea. Journal of Food Composition and Analysis, 12, 211–217.

[fsn3531-bib-0029] Missbach, B. , Schwingshackl, L. , Billmann, A. , Mystek, A. , Hickelsberger, M. , Bauer, G. , … Waldron, K. (2001). In vitro protein digestibility and physicochemical properties of dry red bean (Phaseolus vulgaris) flour: Effect of processing and incorporation of soybean and cowpea flour. Journal of Agricultural and Food Chemistry, 49, 2465–2471. 11368621 10.1021/jf0011992

[fsn3531-bib-0501] Njintang, N. , Waldron, K. , & Mbofung, C. (2001). In vitro protein digestibility and physicochemical properties of dry red bean (Phaseolus vulgaris) flour: effect of processing and incorporation of soybean and cowpea flour. Journal of Agricultural And Food Chemistry, 49, 2465–2471. 11368621 10.1021/jf0011992

[fsn3531-bib-0030] Okpala, L. C. , & Chinyelu, V. A. (2011). Physicochemical, nutritional and organoleptic evaluation of cookies from pigeon pea (Cajanus cajan) and cocoyam (Xanthosoma sp) flour blends. African Journal of Food, Agriculture, Nutrition and Development, 11, 5431–5443.

[fsn3531-bib-0031] de la Rosa‐Millán, J. , Pérez‐Carrillo, E. , & Guajardo‐Flores, S. (2017). Effect of germinated black bean cotyledons (Phaseolus vulgaris L.) as an extruded flour ingredient on physicochemical characteristics, in vitro digestibility starch, and protein of nixtamalized blue maize cookies. Starch ‐ Staerke, 69, 1–10.

[fsn3531-bib-0032] Sathe, S. , Salunke, D. K. , & Cheryan, M. (1984). Technology of removal of unwanted components of dry beans. Critical Reviews in Food Science, 21, 263–287. 10.1080/10408398409527402 6391824

[fsn3531-bib-0033] Sessa, D. J. , & Rackis, J. J. (1977). Lipid‐derived flavors of legume protein products. American Oil Chemist's Society, 54, 468–473.

[fsn3531-bib-0034] Shin, D. , Kim, W. , & Kim, Y. (2013). Physicochemical and sensory properties of soy bread made with germinated, steamed, and roasted soy flour. Food Chemistry, 141, 517–523. 23768388 10.1016/j.foodchem.2013.03.005

[fsn3531-bib-0035] Siddiq, M. , Kelkar, S. , Harte, J. , Dolan, K. , & Nyombaire, G. (2013). Functional properties of flour from low‐temperature extruded navy and pinto beans (Phaseolus vulgaris L.). LWT‐Food Science and Technology, 50, 215–219.

[fsn3531-bib-0036] Simons, C. W. , Hall, C. III , & Biswas, A. (2017). Characterization of pinto bean high‐starch fraction after air classification and extrusion. Journal of Food Processing and Preservation, https://doi.org/10.1111/jfpp.13254.

[fsn3531-bib-0037] Simons, C. W. , Hall, C. III , & Tulbek, M. (2012). Effects of extruder screw speeds on physical properties and in vitro starch hydrolysis of precooked pinto, navy, red, and black bean extrudates. Cereal Chemistry, 89, 176–181.

[fsn3531-bib-0038] Simons, C. W. , Hall, C. III , Tulbek, M. , Mendis, M. , Heck, T. , & Ogunyemi, S. (2014). Acceptability and characterization of extruded pinto, navy and black beans. Journal of the Science of Food and Agriculture, 95, 2287–2291. 25298221 10.1002/jsfa.6948

[fsn3531-bib-0039] Singh, J. , & Whelan, K. (2011). Limited availability and higher cost of gluten‐free foods. Journal of Human Nutrition and Dietetics, 24, 479–486. 21605198 10.1111/j.1365-277X.2011.01160.x

[fsn3531-bib-0040] Stevens, L. , & Rashid, M. (2008). Gluten‐free and regular foods: A cost comparison. Canadian Journal of Dietetic Practice and Research, 69, 147–150. 18783640 10.3148/69.3.2008.147

[fsn3531-bib-0041] Tharanathan, R. N. , & Mahadevamma, S. (2003). Grain legumes – A boon to human nutrition. Trends Food Science and Technology, 14, 507.

[fsn3531-bib-0042] Tharise, N. , Julianti, E. , & Nurminah, M. (2014). Evaluation of physico‐chemical and functional properties of composite flour from cassava, rice, potato, soybean and xanthan gum as alternative of wheat flour. International Food Research Journal, 21, 1641–1649.

[fsn3531-bib-0043] Tosh, S. M. , & Yada, S. (2010). Dietary fibres in pulse seeds and fractions: Characterization, functional attributes, and applications. Food Research International, 43, 450–460.

[fsn3531-bib-0044] Urbano, G. , Aranda, P. , Vílchez, A. , Aranda, C. , Cabrera, L. , Porres, J. M. , & López‐Jurado, M. (2005). Effects of germination on the composition and nutritive value of proteins in Pisum sativum, L. Food Chemistry, 93, 671–679.

[fsn3531-bib-0045] Yamsaengsung, R. , Berghofer, E. , & Schoenlechner, R. (2012). Physical properties and sensory acceptability of cookies made from chickpea addition to white wheat or whole wheat flour compared to gluten‐free amaranth or buckwheat flour. International Journal of Food Science and Technology, 47, 2221–2227.

[fsn3531-bib-0046] Zucco, F. , Borsuk, Y. , & Arntfield, S. D. (2011). Physical and nutritional evaluation of wheat cookies supplemented with pulse flours of different particle sizes. LWT‐Food Science and Technology, 44, 2070–2076.

